# Treatment-seeking for vaginal fistula in sub-Saharan Africa

**DOI:** 10.1371/journal.pone.0216763

**Published:** 2019-11-01

**Authors:** Samson Gebremedhin, Anteneh Asefa

**Affiliations:** 1 School of Public Health, Addis Ababa University, Addis Ababa, Ethiopia; 2 School of Public Health, Hawassa University, Hawassa, Ethiopia; Liverpool School of Tropical Medicine, UNITED KINGDOM

## Abstract

**Background:**

There is dearth of data regarding the treatment-seeking practice of women living with vaginal fistula. The paper describes the health-seeking behaviour of fistula cases in the sub-Saharan Africa (SSA) where the burden of the problem is high.

**Methods:**

The data of 1,317 women who ever experienced fistula-related symptom were extracted from 16 national Demographic and Health Surveys carried out in SSA between 2010 and 2017. The association between treatment-seeking and basic socio-demographic characteristics was analysed via mixed-effects logistic regression and the outputs are provided using adjusted odds ratio (AOR) with 95% confidence intervals (CI).

**Results:**

Among all women who had fistula-related symptom, 67.6% encountered the problem soon after delivery, possibly implying obstetric fistula. Fewer identified sexual assault (3.8%) and pelvic surgery (2.7%) as the underlying cause. In 25.8% of the cases clear-cut causes couldn’t be ascertained and, excluding these ambiguous causes, 91.2% of the women possibly had obstetric fistula. Among those who ever had any kind of fistula, 60.3% (95% CI: 56.9–63.6%) sought treatment and 28.5% (95% CI: 25.3–31.6%) underwent fistula-repair surgery. The leading reasons for not seeking treatment were: unaware that it can be repaired (21.4%), don’t know where to get the treatment (17.4%), economic constraints (11.9%), the fistula healed by itself (11.9%) and feeling of embarrassment (7.9%). The regression analysis indicated, teenagers as compared to adults 35 years or older [AOR = 0.31 (95% CI: 0.20–47)]; and women without formal education compared to women with formal education [AOR = 0.69 (95% CI: 0.51–0.93)], had reduced odds of treatment-seeking. In 25.9% of the women who underwent fistula-repair surgery, complete continence after surgery was not achieved.

**Conclusion:**

Treatment-seeking for fistula remains low and it should be improved through addressing health-system, psycho-social, economic and awareness barriers.

## Background

Vaginal fistula is an abnormal communication between vagina and adjacent tubular structures–usually bladder and rectum–leading to continuous leakage of urine or faeces through the vagina [1]. While vaginal fistula has multiple causes, the foremost aetiology leading to more than 90% of the global burden of fistula is injury secondary to prolonged obstructed labour. This is commonly referred to as obstetric fistula [2]. Other less frequent causes include sexual assault, iatrogenic surgical damage, malignancies and radiation [1].

Vaginal fistula is a debilitating condition that has pervasive consequences on women’s psychological, social, physical and economic wellbeing. Without surgical intervention women with fistula face lifetime embarrassment, isolation, social stigmatization and marital separation [3–5]. Furthermore, they are prone to develop chronic vaginal and urinary tract infections, renal failure, pelvic inflammatory disease and amenorrhea [1,6].

The World Health Organization (WHO) estimated that annually 50,000 to 100,000 women worldwide develop obstetric fistula. While the problem had already been eliminated in the western world, up to three million women in sub-Saharan Africa (SSA) and Asia, are suffering from obstetric fistula [7,8]. The lifetime prevalence of vaginal fistula in SSA is as high as 3 cases/1,000 women of reproductive age and the figure exceeds 5 cases/1,000 women in many countries including Uganda, Kenya, Ethiopia and Tanzania [2].

The most effective way to treat vaginal fistula is surgical closure of the defect [1]. However, in many high-burden countries, access to skilled professionals capable of repairing fistula remains limited and very few hospitals are providing the service. Further, as most of fistula cases are from impoverished and marginalized segments of population, treatment-seeking is likely to be hindered by psychosocial, economic and other contextual factors [9].

Several facility-based qualitative studies and crude estimates suggested that timely treatment-seeking for fistula is generally low and globally as high as two million fistula cases remain untreated [6,10–12]. Yet, empirical quantitative evidence is limited possibly due to the reason that vaginal fistula is a rare event in statistical sense and recruiting adequate cases through community-based surveys is usually impracticable. Recently, many national Demographic and Health Surveys (DHS) surveys have collected treatment-seeking related information in many developing countries but the findings have not been reported possibly due inadequacy of sample size.

This study pools cases from multiple and recent community-based DHS conducted in the SSA region and describes treatment-seeking practices at regional level. Secondary indicators including reasons for not seeking care and success rate for fistula-repair surgery are also presented. While previous studies have suggested that timely treatment seeking for fistula is generally low, the current study provided a concrete and numeric estimate. Furthermore, the it has identified patient-side treatment-seeking barriers that would be relevant while designing and implementing fistula management services and programs in the sub-continent.

## Methods

### Study design and eligibility criteria

**Fig 1:**
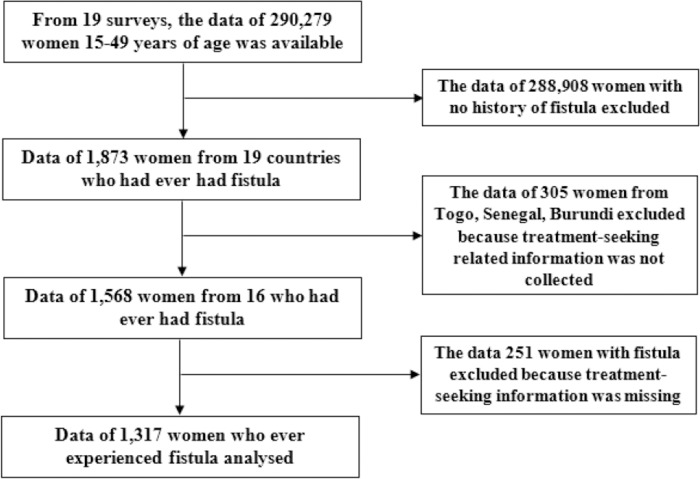
Flow chart of the study.

The study was conducted based on the secondary data of 16 DHS carried in the SSA region between 2010 and 2017. Nineteen surveys that gathered fistula-related information were considered eligible. However, three surveys (Togo 2013–14, Senegal 2017, Burundi 2016–17) did not collect treatment-seeking related data and hence excluded from the analysis. The final dataset comprised the data of 1,317 women with fistula from 14 different countries (Fig 1).

### Sample size and sampling technique

As the study was conducted based on secondary data, priory sample size determination was not made. However, the available number of cases (n = 1,317) is considered sufficient to determine the proportion of treatment seeking for vaginal fistula (68%) with 95% confidence level, 4% margin of error and design effect of 2. Across the countries, the unweighted sample size varied from 13 in Burkina Faso to 246 in Uganda (S1 Table).

The DHS identified eligible subjects (women 15–49 years of age) using two-stage multiple cluster sampling approach. Initially a random sample of primary sampling units (enumeration areas) were drawn and in each unit 20–30 households were selected using simple random sampling approach. Ultimately, all eligible women in the selected households, irrespective of their marital status or birth experience, got interviewed [13].

### Data collection

The primary data were collected by trained enumerators, supervisors and data editors using pretested and standardized questionnaires prepared in the major local languages of the respective countries [13]. In all the surveys the presence of vaginal fistula was assessed by asking all women “did you experience a constant leakage of urine or stool from vagina?”. Then self-reported cause of the leakage was explored and classified as: pregnancy-related, sexual assault, pelvic surgery complications and others. Treatment-seeking practice and reasons for not seeking care were also explored. In most of the surveys, follow up questions whether the problem followed normal or difficult prolonged labour, whether the index delivery ended up in live or stillbirth, were explored. Fistula-related survey questions forwarded in the 16 surveys are provided as a supplementary file (S2 Table).

### Data management and analysis

The datasets of the 16 surveys were separately downloaded from the Measure DHS website and merged into one spreadsheet. Information about women who never experienced fistula was excluded and the remaining data got cleaned and recoded as needed. Data were analysed using the survey data analysis approach via STATA software. Sampling weights provided in the original datasets and post-stratifications weights developed based on the 2017 population size of the countries were used for weighting the data. Wealth index was calculated as the indicator of economic status using the Principal Component Analysis based on ownership of selected household assets. The association between treatment-seeking and basic socio-demographic characteristics (age, place of residence, educational status, wealth index and marital status) was assessed using mixed-effects logistic regression model and the outputs are presented using crude (COR) adjusted odds ratio (AOR) with the respective 95% confidence intervals (CI). The analysed dataset is provided as a supporting file (S1 File).

### Ethical considerations

Ethical clearance has not been sought for this specific analysis. However, the original surveys had been approved by the Institutional Review Board of ORC Macro and national-level ethical committees of the host countries. We accessed the fully anonymized datasets after securing permission from the Measure DHS. In all of the surveys, data were collected after taking informed consent from the study participants.

## Results

### Socio-demographic characteristics

The data of 1,317 women who ever experienced vaginal fistula were analysed. While 16 countries had been represented in the dataset, more than half (60.0%) of the women were from six countries: Uganda, Chad, Malawi, Benin and Sierra Leone. The mean (± SD) age of the women was 30.7 (± 8.9) years and two-thirds (66.0%) were younger than 35 years. Intense reproductive experience appears to be common among the women as nearly one-third (31.2%) had already given birth to five or more children. Further, 40.8% of women were married for the first time before 18 years of age. Table-1 presents the socio-demographic pattern of the study subjects (Table 1).

**Table 1:** Socio-demographic characteristics of women who ever experienced vaginal fistula, 16 sub-Saharan Africa countries, 2010–2017.

Characteristics (n = 1317)	Frequency	Percentage
Place of residence		
Urban	493	37.4
Rural	824	62.6
Age in years		
15–24	367	27.9
25–34	502	38.1
35–49	448	34.0
Educational status		
No formal education	456	34.6
Primary	535	40.6
Secondary	276	20.9
Higher	50	3.8
Marital status		
Never in union	165	12.5
Married/living with partner	972	73.8
Widowed	65	5.0
Divorced	43	3.2
Separated	72	5.5
Total children ever born		
0	153	11.6
1–2	428	32.5
3–4	325	24.7
5 or more	411	31.2
Age at first marriage (n = 1211)		
Before 18 years	475	40.8
18 years or older	689	59.2

### Causes of fistula

Nearly two-thirds (67.6%) of the women reported that the problem of leakage of urine or stool from vagina followed delivery/childbirth possibly implying obstetric fistula. Smaller proportions identified sexual assault (3.8%) and pelvic surgery (2.7%) as the causes of the leakage. In the remaining 25.8% of the cases responses like “don’t know”, “don’t know but did not followed pregnancy” and “others” were given, hence clear-cut causes could not be ascertained. Excluding these ambiguous causes, 91.2% (887 out of 972) of the cases possibly had obstetric fistula (Table 2).

**Table 2:** Reported causes of vaginal fistula, 16 sub-Saharan Africa countries, 2010–2017.

Causes of fistula (n = 1312)	Frequency	Percent
Followed delivery	887	67.6
Sexual assault	50	3.8
Pelvic surgery	36	2.7
Unspecified non-obstetric causes	87	6.6
Others (unspecified)	64	4.9
Don't know	188	14.3

Most of the surveys included in the analysis presented additional information on the leakage that followed pregnancy including time of onset of the leakage and whether the problem followed normal/difficult prolonged labour or not. Accordingly, about a quarter (23.3%) of the leakage started within the first day of birth while nearly half (46.6%) developed it late after one week of birth. In 57.4% of the cases the leakage followed prolonged or very difficult labour suggesting obstetric fistula and 14.1% of the index pregnancies ended up in stillbirths (Table 3).

**Table 3:** Characteristics of fistula that followed pregnancy, 16 sub-Saharan Africa countries, 2010–2017.

Characteristics of obstetric fistula	Frequency	Percent
Onset of leakage (n = 529)		
Within the first day of birth	123	23.3
2–6 days after birth	159	30.1
After the first week of birth	247	46.6
Was the baby born alive (n = 788)		
Yes	663	74.8
No	125	14.1
Problem started after normal or difficult labour? (n = 538)		
Normal labour	229	42.6
Prolonged and very difficult labour	309	57.4

### Health seeking for vaginal fistula

Of the women who had fistula, only 60.3% (95% CI: 56.9–63.6%) sought care from the formal medical sector and 57.6% received treatment. Based on the available information in the dataset, in 1158 of the women it was possible to ascertain whether they had undergone fistula repair-surgery or not. It was found that only 28.5% (95% CI: 25.3–31.6%) women had the surgery. A variety of reasons were reported for not seeking modern treatment. The leading were: lack of the awareness that fistula can be fixed (21.4%), don’t know where to go (17.4%), fear of cost associated with the treatment (11.9%), the problem resolved by itself (11.9%), feeling of embarrassment (7.9%) and inaccessibility of treatment centres (6.5%) (Table 4).

**Table 4:** Health seeking for vaginal fistula, 16 sub-Saharan Africa countries, 2010–2017.

Variables	Frequency	Percentage
Sought care from the formal medical sector (n = 1317)		
Yes	794	60.3
No	523	39.7
Person last sought treatment from (n = 793)		
Health professionals at health facilities	767	96.6
Community health workers	27	3.4
Received any treatment for the fistula (n = 1317)^×^		
Yes	759	57.6
No	558	63.4
Underwent surgery for the fistula (n = 1158)××		
Yes	329	28.5
No	829	71.5
Reasons for not seeking care (n = 468)		
Doesn’t know fistula can be fixed	100	21.4
Treatment is too expensive	84	17.9
Do not know where to go	81	17.4
Problem disappeared by itself	56	11.9
Embarrassment	37	7.9
Treatment facility is too far	31	6.5
Could not get permission	10	2.1
Poor quality of care	6	1.2
Unspecified other reasons	33	7.1

× computed out of all women presented with vaginal fistula

×× 159 women with vaginal fistula had missing data whether they underwent surgery for fistula or not

### Effectiveness of fistula-repair surgery

Among 329 women who underwent fistula-repair surgery, information pertaining to the effectiveness of the treatment was available in the dataset for 244 of them. Of these, 74.1% reported the leak completely stopped after the surgery while the remaining reported that it did not stop but reduced (24.3%) or not stopped at all (1.6%). In aggregate, complete continence after surgery was not achieved in 25.9% of the cases.

### Health seeking behaviour and socio-demographic characteristics

Table 5 presents pattern of health seeking behaviour disaggregated by basic socio-demographic characteristics including current age, place of residence, educational status household wealth index and marital status. Among teenage girls with fistula only 31.5% sought care. As compared to adults 35 years or above, the odds of seeking care among teenage girls were reduced by 69% [AOR = 0.31 (95% CI: 0.20–47)]. Similarly, compared to women with formal education, women with no formal education had 31% reduced odds of seeking care for fistula. No meaningful differences were observed across categories of household wealth index, place of residence and current marital status (Table 5).

**Table 5:** Health seeking behaviour for vaginal fistula disaggregated by basic socio-demographic variables, 16 sub-Saharan Africa countries, 2010–2017.

Variables	% who sought care	Odds Ratio	
		Crude	Adjusted
Current age (years)			
15–19	31.5	0.36 (0.24–0.54)*	0.31 (0.20–0.47)*
20–34	63.4	0.90 (0.71–1.16)	0.86 (0.67–1.11)
35 or above	63.2	1	1
Household wealth index			
Poorest or poorer	56.9	0.75 (0.58–0.97)*	0.90 (0.66–1.21)
Middle	60.3	0.79 (0.58–1.08)	0.85 (0.60–1.20)
Richer or Richest	61.4	1	1
Current educational status			
No formal education	51.8	0.75 (0.57–0.98)*	0.69 (0.51–0.93)*
Any formal education	64.7	1	1
Current place of residence			
Urban	62.6	1	1
Rural	58.9	0.73 (0.57–0.94)*	0.82 (0.60–1.11)
Marital status			
Married/living together	61.3	1	1
Others	57.2	0.90 (0.70–1.16)	0.75–1.32)

* Statistically significant association at p-value of 0.05.

## Discussion

This secondary data analysis indicated that less than two-thirds of all vaginal fistula cases in SSA sought treatment and about a quarter underwent repair surgery. Treatment-seeking is constrained by multiple reasons including lack of awareness about the existence of treatment, feeling of embarrassment, and financial and geographical inaccessibility to treatment centres. Furthermore, health seeking is exceptionally low among teenagers and woman with no formal education. The study also found that in a quarter of women who had fistula-repair surgery, complete continence had not been attained.

We found that only 60% of fistula patients sought treatment from the formal medical sector and a smaller proportion (29%) underwent surgery. The gap between the two figures suggests that the existing medical system is not capable of providing repair surgeries even to known cases. Previous studies in the region reported that the system failed to provide prompt treatment to registered cases due to lack of treatment centres, scarcity of skilled professionals and existence of huge backlogs awaiting treatment [14–16]. A study in Somalia indicated 45% of women could not be able to undergo surgery within one year of registration [14] and in Burkina Faso women had to wait as long as five years to get operated [16].

The leading reasons for failing to seeking treatment for fistula include lack of awareness about the existence of treatment or where to get the treatment, economic constraints, feeling of embarrassment and inaccessibility of treatment centres. Likewise, a systematic review identified psychosocial, cultural, awareness, financial, and transportation domains as the critical barriers to fistula treatment in low income countries [12]. A study based on data from 20 countries concluded physical and financial inaccessibility of treatment sites and low awareness about existing treatment options as the key barriers to treatment seeking [16]. The finding implies that making fistula treatment facilities physically and economically accessible to the population and disseminating information about the curability of the problem through mass media and interpersonal channels may help to promote treatment-seeking behaviour for vaginal fistula.

Unequivocal evidence exists that teenage pregnancy is associated with increased risk of obstetric fistula [17]. This study has indicated that treatment seeking is also exceptionally low (32%) among teenagers living with fistula. To the best of our knowledge, no study has explored the relationship between age and treatment-seeking for fistula. However, multiple studies have witnessed that adolescent girls are less likely to seek for basic maternity services than their adult counterparts due to multiple factors including lower socio-economic status, limited media exposure, influence from others like husbands. Further, teenage pregnancy frequently happens in rural areas were access to fistula treatment is limited [18,19]. The finding suggests that targeting teenage mothers in the promotion of treatment seeking for fistula is critically important for improving coverage of treatment.

The study suggested that women with no formal education have significantly lower odds of seeking care for vaginal fistula as compared to those who had formal education. The observed association can be due to the reason that women with no formal education are likely to have low economic status, tend to be economically dependent on others and may lack household decision making power to seek care for medical problems including fistula. Further, low educational status may limit women’s awareness about the existence of treatment for fistula.

According to the WHO’s proposed indicators for monitoring and evaluating the quality fistula-repair surgery, closure rate should be 85% and complete continence should be achieved in 90% of women with a closed fistula [9]. Yet, we found that only 74% of women attained complete continence after surgery. The lower success rate may suggest the sub-optimal quality of the surgical care or presence of other contextual factors that may limit surgical closure (e.g malnutrition) in the region. Facility-based studies conducted in Ethiopia, Rwanda, Nigeria and Guinea reported complete continence rates range between 83 and 89% [20–23]. However, the figures cannot be directly compared to each other due to multiple reasons including differences in treatment success ascertaining approaches. Further, most facility-based studies evaluate closure rate at discharge but the current study accounted for treatment failure or recurrence that occurred any time after discharge.

One key limitation of the study is that, the DHS assessed the occurrence of fistula among women by asking “did you experience a constant leakage of urine or stool from vagina?” without any further clinical examination. Accordingly, there is a possibility for the women to misreport other medical conditions like postpartum urethral stress incontinence or faecal inconsistence as obstetric fistula. Such misclassification errors might have caused underestimation of key indicators presented in this article including magnitude of treatment seeking and repair surgery for fistula. On the other hand, a recent study in Nigeria has suggested, as compared to medical screening for fistula, self-reported symptoms have reasonably high sensitivity (92%) and specificity (83%) for identifying women affected by fistula [24].

Obstetric fistula usually follows unassisted, prolonged and difficult labour. However, in the current study, among women who encountered postpartum vaginal urinary/faecal leakage, only 57.4% reported prolonged or very difficult labour. This unexpected finding might be due to the misclassification error that we stated above. Furthermore, classifying labour as prolonged/very difficult or normal by the women can be too technical and it is prone to subjectivity. Another limitation of the study is that the DHS questionnaire measured lifetime occurrence of fistula and does not specifically tell when the incident actually happened. Consequently, recall errors are possible and indicators like treatment-seeking practice and surgical success rate presented in this paper may not exactly indicate the contemporary situations in the region. Assuming access to treatment and quality of surgical care have improved over years, the study might have underrated the aforementioned two indices. Furthermore, the DHS did not measure the number of times the women had experienced fistulae.

In addition, measurement of the association between current socio-demographic variables (e.g. current educational status and wealth status) and treatment-seeking which happened sometime in the past bears an erroneous assumption that socio-demographic variables are fixed and this leads to an obvious underestimation of the strength of association between the variables. Another key limitation is that the study entirely used quantitative methods and no qualitative approach was employed to understand socio-cultural and other contextual factors that may hinder/facilitate the health-seeking behaviour of the women to vaginal fistula.

Caution should also be taken while generalizing the findings of the study to the entire SSA region because the health system and socio-demographic profile of the represented 16 countries may not be exactly the same as that of the other countries in the region. It is also important to note that many DHS conducted between 2010 and 2017 in the region did not collected fistula-related information partly because the respective countries did not have considered fistula as their priority problem. This suggests that the represented 16 countries are systematically different from the remaining SSA countries. However, we believe the study provides a reasonable picture of the treatment-seeking behaviour in the sub-continent.

## Conclusion

In the SSA region, treatment-seeking for fistula is low and is constrained by multiple factors–both from the health system and the patients’ perspectives. Women living with fistula have low treatment-seeking behaviour due to lack of awareness, sense of embarrassment as well as financial and physical inaccessibility to treatment centres. Further, the existing medical system is incompetent even to provide repair surgery to known cases. Teenage girls and women devoid of formal education are less likely to seek care for fistula treatment.

In the region, treatment-seeking should be augmented via a mix of strategies for abridging health-system, psycho-social, economic and awareness barriers. Fistula treatment facilities should be more physical and economically accessible to the population of the region. Further, information about the curability of the problem should be disseminated though mass-media and interpersonal channels.

## Supporting information

S1 Table Sample size distribution by country. (DOCX)

S2 Table Fistula-related survey questions forwarded in the 16 Demographic and Health Surveys. (XLSX)

S1 File Dataset analysed for the study. (XLSX)
