# Supplementary material for: Treatment-seeking for vaginal fistula in sub-Saharan Africa
Source: PLoS One. 2019 Nov 1;14(11):e0216763. doi: 10.1371/journal.pone.0216763 (PMC6824565; doi:10.1371/journal.pone.0216763)
Supplement: S1 Table — (DOCX) [file pone.0216763.s001.docx]

Supporting file 1: Sample size distribution by country.

| **Country** | **Year of survey** | **Sample size (n=1317)** | |
| --- | --- | --- | --- |
|  |  | **Unweighted** | **Weighted** |
| Burkina Faso | 2010 | 13 | 12 |
| Benin | 2011-12 | 127 | 124 |
| Congo | 2011-12 | 21 | 25 |
| Cameroon | 2011 | 40 | 46 |
| Ethiopia | 2016 | 70 | 69 |
| Guinea | 2012 | 61 | 58 |
| Kenya | 2014 | 86 | 88 |
| Comoros | 2012 | 103 | 76 |
| Mali | 2012-13 | 46 | 42 |
| Malawi | 2015-16 | 146 | 141 |
| Niger | 2012 | 16 | 19 |
| Sierra Leone | 2013 | 103 | 107 |
| Chad | 2014-15 | 150 | 165 |
| Tanzania | 2010 | 24 | 29 |
| Uganda | 2016 | 246 | 254 |
| Zambia | 2013-14 | 65 | 61 |
